# Transcriptomic and epigenetic profiling of neuroblastoma states in response to RBM39 degrader

**DOI:** 10.1038/s41597-025-06408-4

**Published:** 2025-12-09

**Authors:** Hongjian Jin, Jie Fang, Jason Myers, Shivendra Singh, Jun Yang

**Affiliations:** 1 Center for Applied Bioinformatics, St Jude Children’s Research Hospital, 262 Danny Thomas Place, Memphis, Tennessee 38105 USA; 2 Department of Surgery, St Jude Children’s Research Hospital, 262 Danny Thomas Place, Memphis, Tennessee 38105 USA; 3 Department of Pathology and Laboratory Medicine, College of Medicine, The University of Tennessee Health Science Center, 930 Madison Ave, Suite 500, Memphis, TN 38163 USA; 4 College of Graduate Health Sciences, University of Tennessee Health Science Center, Memphis, TN 38163 USA

**Keywords:** Embryonal neoplasms, Tumour heterogeneity

## Abstract

Neuroblastoma accounts for approximately 15% of all pediatric cancer-related deaths, largely due to disease relapse following intensive multimodal therapy. A critical barrier to cure neuroblastoma is the emergence of therapy-resistant tumor cells. Neuroblastoma comprises two major cell states, adrenergic (ADRN) and mesenchymal (MES), which are believed to interconvert and contribute to therapeutic resistance through lineage plasticity. To investigate the mechanisms underlying this plasticity, we subjected human and murine neuroblastoma models to repeated treatment with indisulam, a molecular glue compound that selectively degrades the splicing factor RBM39, until full drug resistance emerged. We then generated datasets from these models, including bulk transcriptomic data, ATAC-seq, and H3K27ac CUT&Tag. These data comprehensively characterize transcriptomic and epigenetic landscapes of resistant ADRN and MES neuroblastoma cell states. We present this resource to facilitate reuse by the scientific community. These datasets may support efforts to decipher lineage switching, identify regulators of therapy resistance, and discover potential therapeutic vulnerabilities in resistant neuroblastoma.

## Background & Summary

Lineage plasticity confers cancer cell ability to acquire alternative cellular state in response to therapies, ultimately leading to drug resistance^1–5^. Neuroblastoma is a sympathetic nervous system malignancy resulting from differentiation arrest of neural crest−derived sympathoadrenal progenitor cells^6,7^, transformed by MYC oncogenes^6,8–10^. Due to refractory or relapsed disease, the 5-year survival rate for patients with high-risk neuroblastoma is only about 50%^11–14^, even with current intensive multimodal therapies (combined chemotherapies, stem cell transplantation, radiotherapy and anti-GD2–based immunotherapy). Neuroblastoma cells contain morphological variants that are composed of two major cellular states, committed adrenergic (ADRN) and neural crest migratory or mesenchymal (MES), defined by distinct epigenetic and transcriptomic programs^15,16^. These distinct populations exhibit differential responses to chemotherapy^16^, leading to the hypothesis that lineage interconversion between ADRN and MES states is one of the underlying mechanisms accounting for chemoresistance of high-risk neuroblastomas.

While MES and ADRN states were presumed to be interconvertible, this was largely supported by one direction conversion from ADRN to MES through forced overexpression of lineage-specific MES transcription factors such as PRRX1 and NOTCH or genetic deletion of ARID1A in cell lines^16–18^. However, evidence to support the conversion from MES to ADRN was scarce although one study indicates that spontaneous conversion could occur *in vivo* when a MES state neuroblastoma cell line was implanted into mice^19^. Nevertheless, it was less clear about the roles of interconversion of ADRN and MES in chemoresistance, particularly under the *in vivo* settings. Distinct from cell lines that have two distinct cell states, nearly all neuroblastomas were classified as ADRN^19^, albeit increased MES signatures and reduced ADRN signatures were seen in high-risk neuroblastomas. Further, ADRN tumors with MES features have molecular traits of Schwann cell progenitors (SCP), bridge cells and early neuroblasts^6,7^, all of which are neural crest cell progenies, indicating those tumors had intratumoral plasticity that may not be only confined to ADRN and MES states. While these studies suggest cell plasticity may be an intrinsic feature of high-risk neuroblastomas and associated with therapy resistance, it was challenging to directly verify the assumptions that neuroblastoma cells switch lineage in bi-direction or multi-direction in response to drug treatments.

**Fig. 1:**
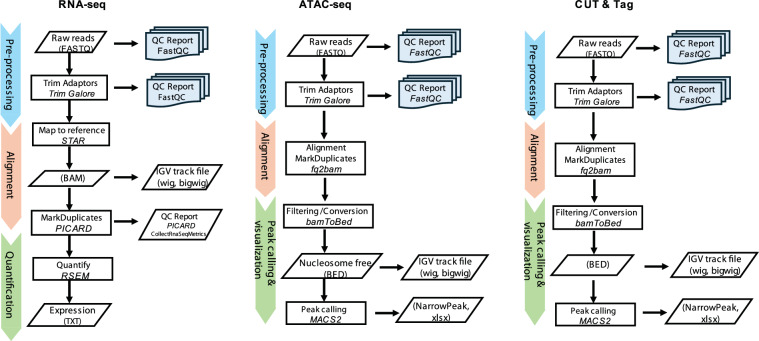
Schematic overview of workflows of data processing pipelines including RNA-seq pipeline, ATAC-seq pipeline, and CUT&Tag pipeline. Tools and algorithms are indicated in italic text. Processed output file formats are enclosed in parentheses.

We recently identified the pre-mRNA splicing factor RBM39 as a new therapeutic target of neuroblastoma^20^. We and others further found that indisulam, a “molecular glue” drug that selectively degrades RBM39 protein^21,22^, was exceptionally effective against neuroblastoma^20,23^. Nevertheless, tumors eventually relapsed in immune-deficient mice despite the complete tumor response. This phenotype mimicked the clinical response of some high-risk neuroblastomas to combined chemotherapy. By taking advantage of these findings, we generated indisulam-resistant tumors after repeated treatments by using *Th-MYCN/ALK*^*F1178L*^ transgenic mouse models (ADRN), SJNB14 patient-derived xenograft (PDX) models (*MYCN* amplified, ADRN), SIMA xenografts, and SK-N-AS cell line-based xenografts (c-MYC overexpressed, MES)^24^. We performed RNA-seq, ATAC-seq and H3K27Ac CUT&Tag to characterize the transcriptomic and epigenetic profiling of naïve vs indisulam-resistant tumors^24^ (Fig. 1). In this Data descriptor, we provided details for these datasets to facilitate their reusing in benchmarking of computational tools for differential expression or splicing analysis, cross-dataset comparison of drug resistance mechanisms and lineage plasticity programs across cancer types, and validation of machine learning models predicting drug response or transcriptional states.

## Methods

### Animal ethics statement

All research was conducted in accordance with relevant ethical guidelines. Animal procedures were reviewed and approved under the protocol 615 issued to Jun Yang in accordance with the guidelines outlined by the St Jude Children’s Research Hospital Institutional Animal Care and Use Committee (IACUC). Mice were housed with ambient temperature and humidity with 12 h light /12 h dark cycle controlled under specific-pathogen-free conditions (SPF) at the St Jude Children’s Research Hospital mouse facility. Mice were allowed to feed and drink ad libitum. The maximal tumor burden permitted was 20% of mouse body weight.

### RNA-seq protocol

Total RNA from SK-N-AS cells (2 × 106 cells per sample) and tumor tissues of SIMA and SJNB14 xenografts and *Th-MYCN/ALK*^*F1178L*^ genetic mouse model (~30 mg) were performed using the RNeasy plus Mini Kit (Qiagen, 74136) according to the manufacturer’s instructions. RNA quality was checked by RNA ScreenTape assay.

### ATAC-seq protocol

Library preparations for ATAC-seq were based on a published protocol with minor modifications. Briefly, freshly cultured SKNAS cells (100,000 per sample, naïve and two resistant ones) were harvested and washed with 150 µl ice-cold Dulbecco’s Phosphate-Buffered Saline (DPBS) containing protease inhibitor (PI). Nuclei were collected by centrifugation at 500 g for 10 minutes at 4 °C after cell pellets were resuspended in lysis buffer (10 mM Tris-Cl pH 7.4, 10 mM NaCl, and 3 mM MgCl_2_ containing 0.1% NP-40 and PI). Nuclei were incubated with Tn5 transposon enzyme in transposase reaction mix buffer (Illumina) for 30 min at 37 °C. DNAs were purified from the transposition sample by using Mini-Elute PCR purification kit (Qiagen, 28004) and measured by Qubit. Polymerase chain reaction (PCR) was performed to amplify with High-Fidelity 2X PCR Master Mix [72 °C/5 mins + 98 °C /30 s + 12 × (98 °C /10 s + 63 °C/30 s + 72 °C/60 s) + 72 °C/5 min]. The libraries were purified using the Mini-Elute PCR purification kit (Qiagen, 28004).

### H3K27Ac CUT&Tag protocol

CUT&Tag DNAs from SKNAS-naïve, IR1 (indisulam-resistant 1) and IR2 (indisulam-resistant 2) cells and SJNB14 PDX-ctrl (control, named as NB14-ctrl) and SJNB14 PDX-IR (indisulam-resistant, named as NB14-IR) tumors were prepared by following the protocol as described previously (Kaya-Okur *et al*.^25^ and https://www.protocols.io/view/bench-top-cut-amp-tag-bcuhiwt6?step=1) with minor modifications. Briefly, for NB14-ctrl and NB14-IR tumor, nuclei extraction is following the protocol of isolate nuclei from frozen tissues (https://support.missionbio.com/hc/en-us/article_attachments/4421562098967). For SKNAS-naïve, IR1 and IR2 cells were washed with wash buffer (20 mM HEPES pH 7.5; 150 mM NaCl; 0.5 mM Spermidine; 1 × Protease inhibitor cocktail). Nuclei were isolated with cold NE1 buffer (20 mM HEPES–KOH, pH 7.9; 10 mM KCl 0.1%; Triton X-100; 20% Glycerol, 0.5 mM Spermidine; 1x Protease Inhibitor) for 10 min on ice. Nuclei were collected by 600 × g centrifuge and resuspended in 1 ml washing buffer containing with 10 µL of activated concanavalin A-coated beads (Bangs laboratories, BP531) at RT for 10 min. Bead-bound nuclei were collected by placing tube on magnet stand and removing clear liquid. The nuclei bound beads were resuspended in 50 µL Dig-150 buffer (20 mM HEPES pH 7.5; 150 mM NaCl; 0.5 mM Spermidine; 1 × Protease inhibitor cocktail; 0.05% Digitonin; 2 mM EDTA) and incubated with a 1:50 dilution of H3K27ac (Abcam, ab4729) overnight at 4 °C. The unbound primary antibody was removed by placing the tube on the magnet stand and removing the liquid. The primary antibody bound nuclei beads were mixed with Dig-150 buffer 100uL containing guinea pig anti-Rabbit IgG antibody (Antibodies, ABIN101961) in 1:100 dilution for 1 hour at RT. Beads bound nuclei were washed using the magnet stand 3 × for 5 min in 1 mL Dig-150 buffer to remove unbound antibodies. A 1:100 dilution of pA-Tn5 adapter complex was prepared in Dig-300 buffer (20 mM HEPES, pH 7.5, 300 mM NaCl, 0.5 mM Spermidine, 0.05% Digitonin, 1 × Protease inhibitor cocktail). After removing the liquid, 100 µL mixture of pA-Tn5 and Dig- 300 buffer was added to the nuclei bound beads with gentle vortex and incubated at RT for 1 h. After washing 3 × 5 min in 1 mL Dig-300 buffer to remove unbound pA-Tn5 protein, nuclei were resuspended in 250 µL Tagmentation buffer (10 mM MgCl_2_ in Dig-300 buffer) and incubated at 37 °C for 1 h. 10 µL of EDTA (0.5 M), 3 µL of 10% SDS and 2.5 µL of 20 mg/mL Proteinase K were added to stop tagmentation by incubation at 55 °C for 1 hour. DNA libraries were then purified with SPRIselect beads (Beckman Coulter, B23318) following manufacture’s instruction and then dissolved in water.

### Data processing and quality control of RNAseq

We used FastQ Screen v0.14.1 to extract the first 10,000 reads from the clean FASTQ files to detect whether the raw data were contaminated with other species, vector sequences, etc. Then, we used the BBSplit method to remove mouse reads (GRCm38) misaligned to the human genome (GRCh38) in the xenograft samples. After xenograft cleansing, filtered reads were used as input to the standard RNAseq workflow described as follows. Briefly, FastQC v0.11.8 (https://www.bioinformatics.babraham.ac.uk/projects/fastqc/) was used to check the quality of reads before and after trimming adaptors (Trim-Galore v0.63). Trimmed reads were mapped by STAR v2.7.9a and gene level values were quantified (RSEM v1.3.3) with gene models from Gencode Human Release 31. Moreover, transcripts per kilobase of exon model per million mapped reads (TPM) were calculated to normalize the gene expression.Count per million mapped reads (CPM) values were log2 transformed as input for principal component analysis (PCA).

### ATAC-seq analysis

BBSplit was used to remove mouse reads (GRCm38) misaligned to the human genome (GRCh38) in xenograft samples. FastQC v0.11.8 was used to check the quality of reads before and after trimming adaptors. Then trimmed reads were aligned to the human reference genome (GRCh38) and marked duplicated reads using Parabricks 4.3.0 fq2bam, with only high-quality reads kept by samtools v1.3.1, parameter “-q 1 -F 1024”. Reads mapping to mitochondrial DNA were excluded from the analysis. All mapped reads were offset by + 4 bp for the + strand and -5 bp for the – strand. Peaks were called for each sample using MACS2 v2.2.7.1 with parameters “-q 0.01 –nomodel –extsize 200 –shift 100”. Peaks were merged for the same cell types using BEDtools v2.25.0. Peak annotation was performed using HOMER. All sequencing tracks were viewed using the Integrated Genomic Viewer v2.3.82.

### H3K27Ac CUT&Tag analysis

BBSplit was used to extract human (GRCh38) portion of data as input to the standard workflow of CUT&Tag analysis. In short, FastQC v0.11.8 was used to check the quality of reads before and after trimming adaptors. Trimmed reads were aligned to the human reference genome (GRCh38) and marked duplicated reads using Parabricks 4.3.0 fq2bam. Only properly paired uniquely mapped reads were extracted by samtools with parameters of “-q 1 -f 2 -F 1804” for calling peaks and generating bigwig file. Narrow peaks were called by MACS2 v2.2.7.1 with parameters of “ -t cut_tag_file -q 0.05 -f BED–keep-dup all”.

## Data Records

The datasets for RNA-seq, ATAC-seq and CUT&Tag are available at NCBI Gene Expression Omnibus (original: GSE164506^26^, GSE251920^27^; reanalysis: GSE299174^28^).

## Technical Validation

### Quality control of RNA integrity

The quality of total RNA was examined by an Agilent Technologies TapeStation RNA ScreenTape. All of the samples showed high RNA integrity (RIN value ranging over 9) and were used for downstream sequencing.

### RNA-seq quality control

**Fig. 2:**
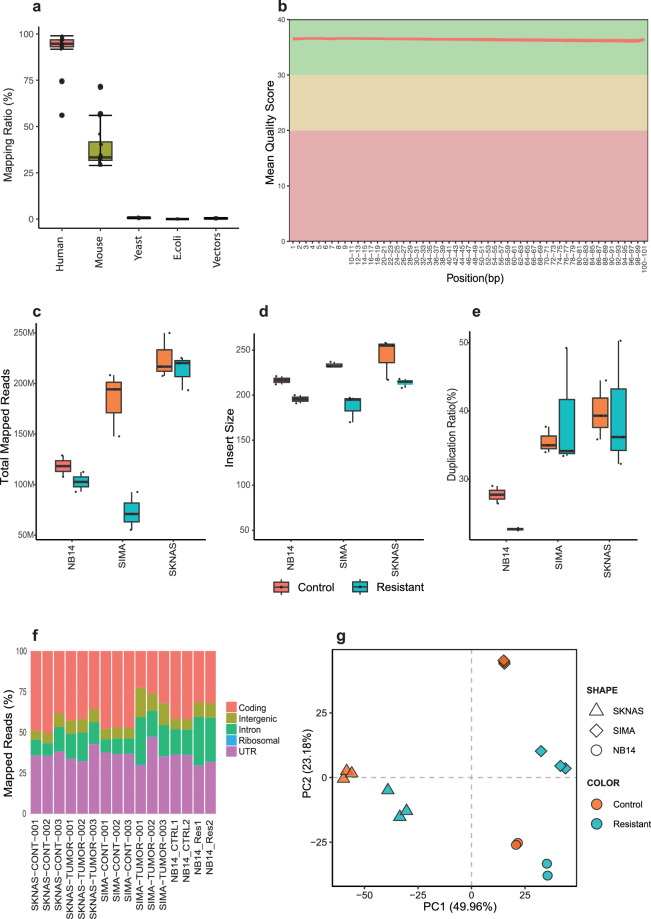
Quality control metrics of RNA-seq data from SK-N-AS, SIMA and SJNB14 (n = 16). (**a**) Box plot representing mapping ratio (%) across all samples to different reference genomes or species by FastQ Screen. (**b**) Line plot presenting an overview of high-quality scores across all bases at each position in the FASTQ file of all samples. Box plots representing the total mapped reads (**c**), median insert size of the data (**d**) and duplication ratio of mapped reads (**e**) across all samples per experimental group.(**f**) Bar plot percentage of reads that map to gene sequence categories across all samples. (**g**) Principal Component Analysis (PCA) of all tumors. Control indicates naïve tumor samples. Resistant indicates indisulam resistant samples.

The quality of RNA-seq data was assessed using FastQ Screen and FastQC. After the QC process, reads were aligned using STAR. The BAM files were generated using Samtools and then were further processed with Picard to mark duplicate reads, check insert size and collect RNA-Seq metrics. FastQ Screen revealed 30%-71% mouse contaminant reads (a median of 33.3%, Fig. 2a, Supplementary Table 1a). After cleansing contaminant reads, a series of QC metrics RNA-seq were generated (Supplementary Table 1b). The overall RNA-seq dataset was of high quality:

1. the Phred quality scores across all bases at each position in the FASTQ file of all samples were consistently high (>35) (Fig. 2b);
2. the average mapped reads of all samples were around 157 millions (Fig. 2c);
3. the average length of insert size was approximately 216 bp (Supplementary Table 1b, Fig. 2d);
4. the average duplication rate of mapped reads from all samples was around 35% (Fig. 2e, Supplementary Table 1b);
5. the distribution of gene region occupancy was consistently high (Fig. 2f).

**Fig. 3:**
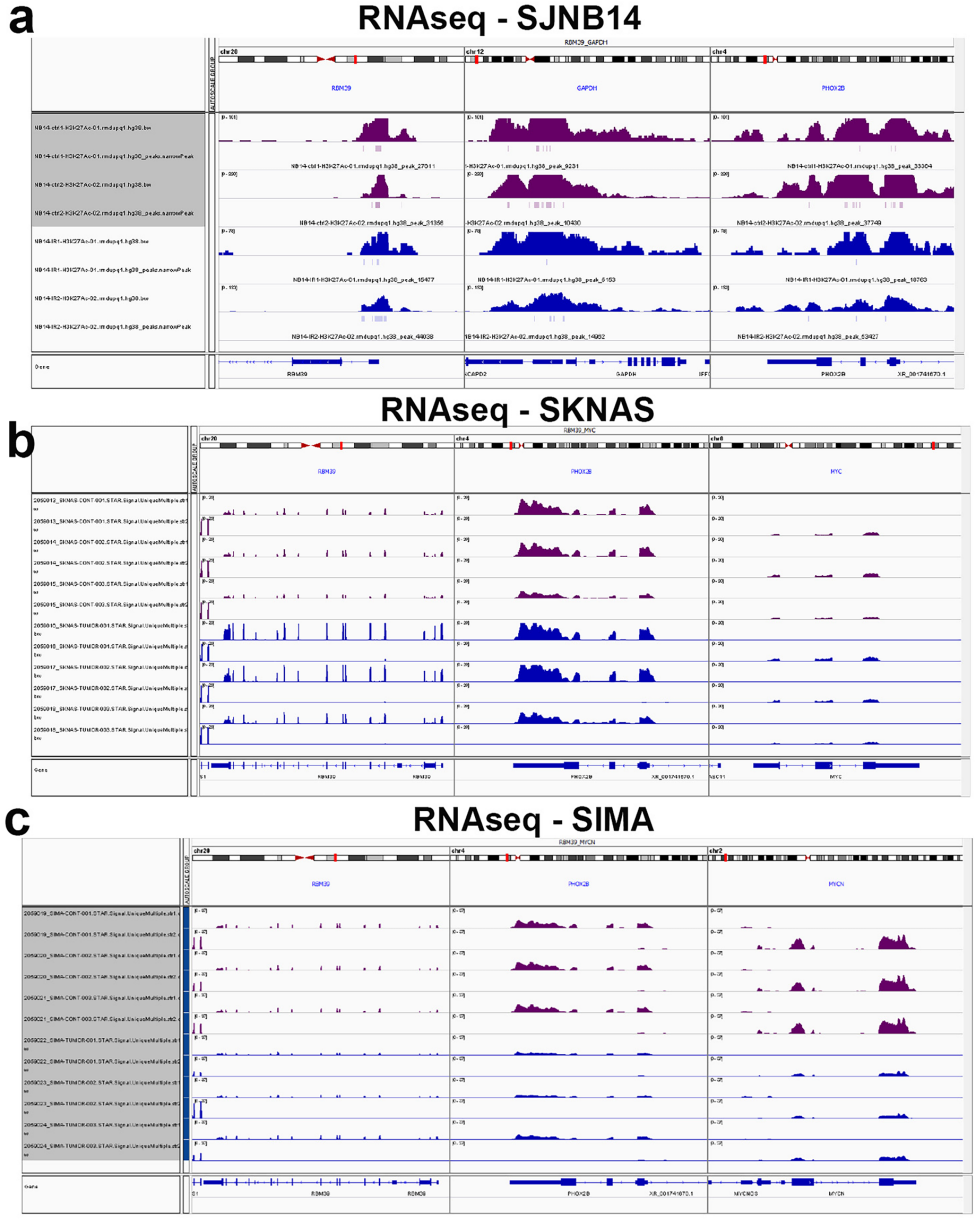
IGV tracks showing RNA-seq read peaks at representative loci. RNA-seq IGV tracks at the *RBM39*, *GAPDH* and *PHOX2B* in SJNB14 (**a**), *RBM39*, *PHOX2B* and *MYC i*n SKNAS (**b**), *RBM39*, *PHOX2B* and *MYCN i*n SIMA (**c**) tumors, respectively. Magenta color indicates naïve tumors. Dark blue indicates indisulam-resistant tumors.

Moreover, the principal component analysis (PCA) results showed that resistant and the paired control samples can be well separated at the first principal component (Fig. 2g), indicating good quality of RNA-seq data at the expression level. RNA-seq peaks can be visualized by Integrative Genomics Viewer (IGV) as exemplified (Fig. 3)

### ATAC-seq quality control

**Fig. 4:**
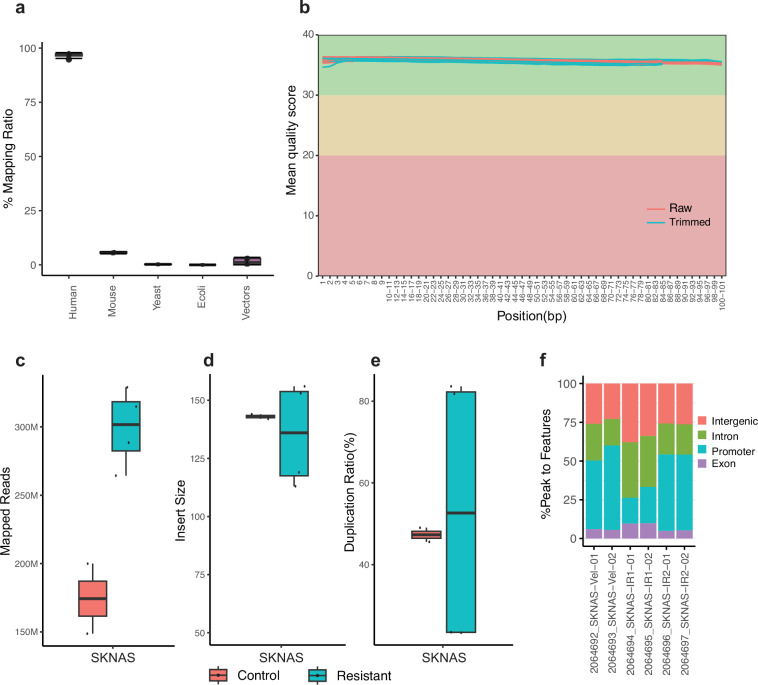
Quality control metrics of ATAC-seq data (n = 6) derived from SK-N-AS samples. (**a**) boxplot visualizing the distribution of read mapping results by FastQ Screen to different reference genomes or species. Box plot representing mapping ratio (%) across all samples of multiple sources. (**b**) Line plot presenting an overview of high-quality scores across all bases at each position in the FASTQ file of all samples. Box plots representing the total mapped reads (**c**), median insert size of the data (**d**), percentage of duplicated reads (**e**) and peak genomic distribution(**f**) across all samples.

**Fig. 5:**
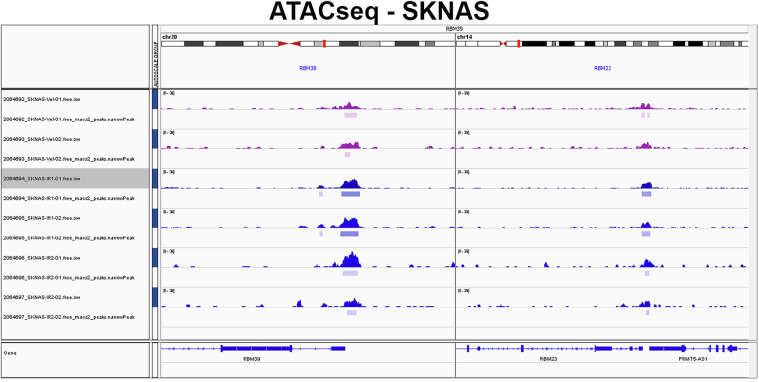
IGV tracks showing ATAC-seq read peaks at the RBM39 and RBM23 loci in naïve SKNAS (magenta color) and indisulam-resistant SKNAS tumors (dark blue).

The quality of ATAC-seq data was assessed using FastQ Screen and FastQC. After the QC process, reads were aligned using BWA. Fastq_screen revealed minimal contaminant reads (<7% reads mappable to mouse genome GRCm38, Fig. 4a, Supplementary Table 2a). The BAM files were generated using Samtools and then were further processed with Picard to mark duplicate reads and check insert size. FastQC quality scores across all bases at each position in the FASTQ file of all samples were consistently high (Fig. 4b). The average mapped reads of all samples were around 257 millions (Fig. 4c, Supplementary Table 2b). The average length of insert size was approximately 143 bp (Fig. 4d, Supplementary Table 2c), and the median duplication rate of mapped reads from all samples was around 50% (Fig. 4e). The genomic distribution for ATAC-seq peaks located in promoter, exon and intron, and intergenic regions was also provided (Fig. 4f). ATAC-seq peaks can be visualized by Integrative Genomics Viewer as exemplified (Fig. 5).

### H3K27Ac CUT&Tag quality control

The quality of CUT&Tag data was assessed using FastQ Screen and FastQC. After the QC process, reads were aligned using BWA. The BAM files were generated using Samtools and then were further processed with Picard to mark duplicate reads and check insert size.

**Fig. 6:**
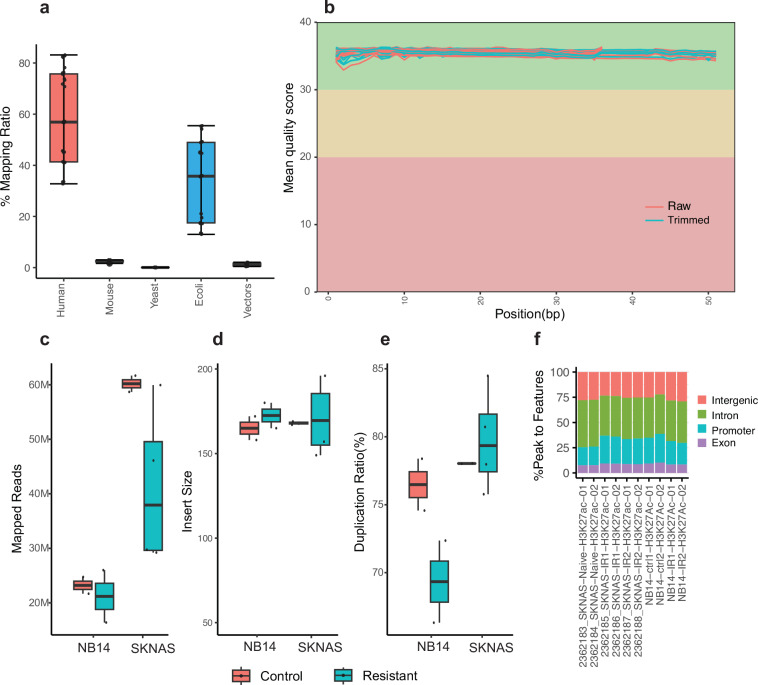
Quality control metrics of CUT&Tag data in both SK-N-AS and SJNB14 (n = 28). (**a**) boxplot visualizing the distribution of read mapping results by FastQ Screen to different reference genomes or species. (**b**) Line plot presenting an overview of high-quality scores across all bases at each position in the FASTQ file of all samples. Box plots representing the total mapped reads (**c**), median insert size of the data (**d**), percentage of duplicated reads (**e**) and peak genomic distribution(**f**) across all samples.

**Fig. 7:**
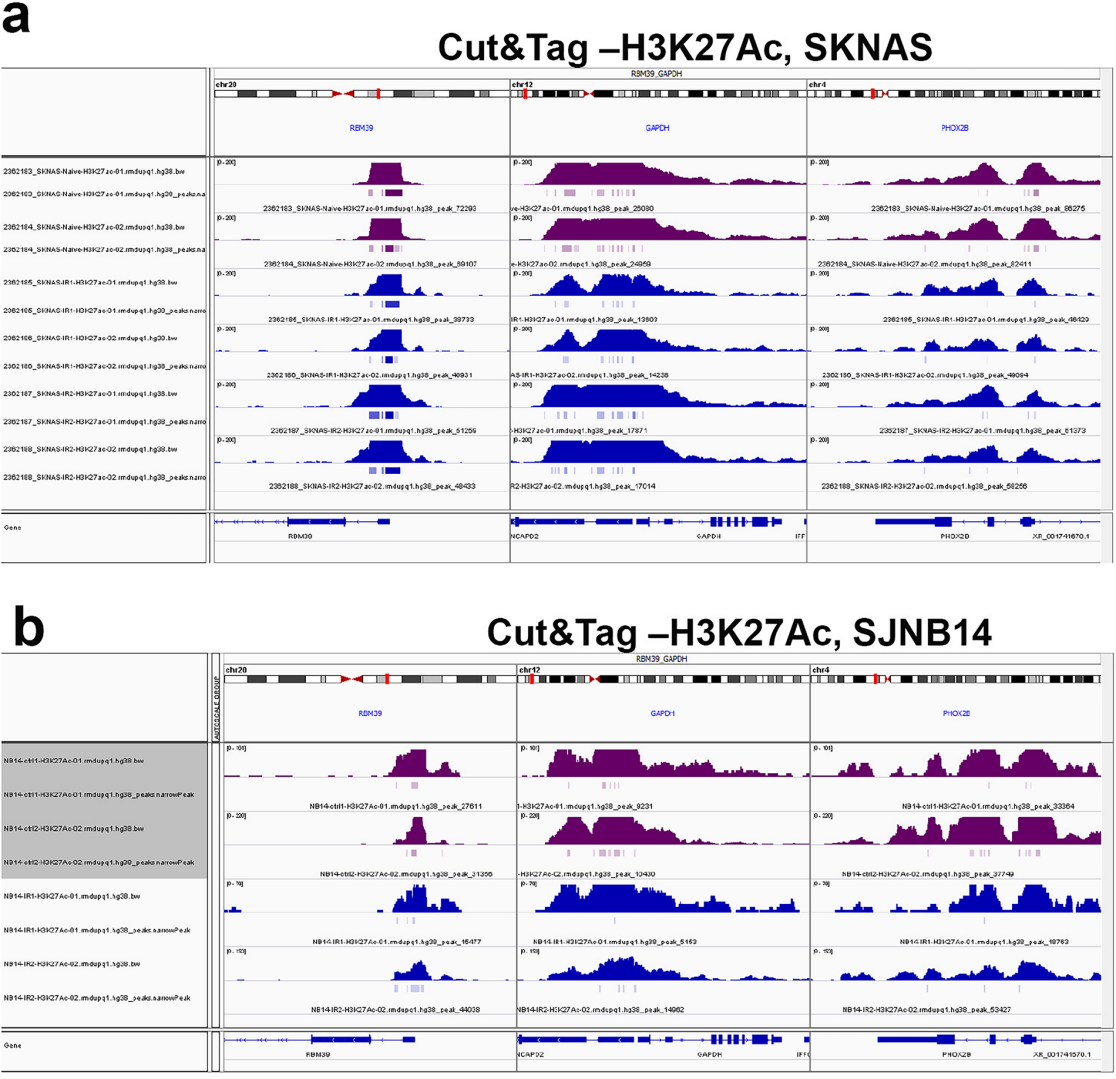
IGV tracks showing H3K27ac CUT&Tag read peaks at the representative loci. **(a)** CUT&Tag read peaks at *RBM39, GAPDH* and *PHOX2B* in naïve SKNAS (magenta color) and indisulam-resistant SKNAS tumors (dark blue). **(b)** CUT&Tag read peaks at *RBM39, GAPDH* and *PHOX2B* in naïve SKNAS (magenta color) and indisulam-resistant SKNAS tumors (dark blue).

According to FastQ Screen results, a median of 33.6% of reads aligned to the *E. coli* reference genome (Fig. 6a, Supplementary Table 3a). FastQC quality scores across all bases at each position in the FASTQ file of all samples were consistently high (>35) (Fig. 6b). The average mapped reads of all samples were around 37 millions (Fig. 6c, Supplementary Table 3b). The average length of insert size was approximately 168 bp (Fig. 6d, Supplementary Table 3c). The median duplication rate of mapped reads from all samples was around 50% (Fig. 6e). The genomic distribution for CUT&Tag peaks located in promoter, exon and intron, and intergenic regions was also provided (Fig. 6f). CUT&Tag peaks can be visualized by Integrative Genomics Viewer (IGV) as exemplified (Fig. 7).

## Supplementary information

Supplementary Table 1

Supplementary Table 2

Supplementary Table 3

## Data Availability

The dataset has been deposited to GEO (GSE299174).

## Code availability

All samples were processed by internal software AutoMapper, which includes all pre-defined NGS mapping pipelines, supported by the Center for Applied Bioinformatics (CAB), St Jude Children’s Research Hospital. The pseudo-codes and detailed parameters of pre-processing pipelines for RNAseq, ATACseq and CUT&Tag data in current study can be found in the GitHub repository (https://github.com/hongjianjin/yang2grp). Quality control metrics for all data were collected in the metadata tables and visualized using R v4.1.0 (https://cran.r-project.org/, R development core team).
